# Universal type/subtype-specific antibodies for quantitative analyses of neuraminidase in trivalent influenza vaccines

**DOI:** 10.1038/s41598-017-18663-6

**Published:** 2018-01-18

**Authors:** Kangwei Xu, Changgui Li, Caroline Gravel, Zheng Jiang, Bozena Jaentschke, Gary Van Domselaar, Xuguang Li, Junzhi Wang

**Affiliations:** 1 The State Key Laboratory of Cancer Biology, Department of Biochemistry and Molecular Biology, The Fourth Military Medical University, Xi’an, Shaanxi 710032 P.R. China; 2 National Institutes for Food and Drug Control and WHO Collaborating Center for Standardization and Evaluation of Biologicals, Beijing, 102619 P.R. China; 3 Center for Biologics Evaluation of Biologicals, Ottawa, Biologics and Genetic Therapies Directorate, Health Canada and WHO Collaborating Center for Standardization and Evaluation, Ottawa, ON K1A 0K2 Canada; 4 National Microbiology Laboratory, Public Health Agency of Canada, Winnipeg, MB R3E 3R2 Canada; 5 Department of Biochemistry, Microbiology and Immunology, Faculty of Medicine, University of Ottawa, Ottawa, ON Canada

## Abstract

Both influenza viral hemagglutinin and neuraminidase can induce protective immune responses in humans. Although the viral hemagglutinin antigens have been quantified in influenza vaccines, the amounts of neuraminidase remain undetermined. Using comprehensive bioinformatics analyses of all neuraminidase sequences, we identified highly conserved and subtype-specific peptide epitopes within each of N1, N2 and type B neuraminidase groups. Mono-specific antibodies generated against these peptides bound to their respective subtype/type only while demonstrating remarkable specificity against the viral neuraminidase sequences without any cross-reactivity with allantoic and cellular proteins. Moreover, the subtype/type-specific antibodies were found not to interfere with one another when a mixture of vaccine samples was analysed. Importantly, immunoassay based on these antibodies can quantitatively determine neuraminidase in commercial trivalent vaccine samples. Analyses of vaccines from eight manufacturers using the same vaccine seeds revealed significant differences in neuraminidase levels. Specifically, while the ratio between neuraminidase and hemagglutinin in some products are found to be close 1/5, other products have a ratio of approximately 1/100, a level which is far below the theoretical ratio between neuraminidase and hemagglutinin in a virus. The antibody-based assays reported here could be of great value for better quality control of both monovalent and trivalent vaccines.

## Introduction

Influenza remains a serious ongoing threat to public health. The WHO estimates between three to five million cases of severe illness and 250,000 to 500,000 deaths per year are caused by influenza virus. Vaccination is the most effective means to prevent influenza. The surface of influenza virus contains two glycoproteins, hemagglutinin (HA) and neuraminidase (NA), both of which are known to induce protective immune responses in human and animals.

HA is the most abundant glycoprotein, which is essential for receptor-binding and fusion of viral membranes with the host cells. Currently, the trivalent inactivated vaccine (TIV) is the most widely used form of vaccine. It is comprised of inactivated viruses derived from one H1N1, one H3N2 and one type B strain. Each of these HAs is updated annually by the WHO. The potency of TIV is determined by traditional single radial immunodiffusion (SRID) using strain specific antibodies generated every year. In contrast, no standardized assay is available for quantification of NA in the final products of trivalent vaccines.

NA is the second major glycoprotein on the viral surface. In type A virus, NA is classified into 9 subtypes whereas NA in type B virus (NB) has not been subtype-classified. It is well established that NA facilitates the release of newly generated virus from the infected cell membrane by cleaving sialic acid from host cell receptor. It also prevents aggregation of viral particles by cleaving the sialic acid residues present on viral glycoproteins. NA-specific immune responses have been shown to protect the host from influenza^1^. Specifically, in preclinical studies, animals immunized with NA antigen can be protected from influenza; NA-specific antibodies are by themselves sufficient to afford protection from lethal challenges^1–8^. Moreover, NA-specific immune responses in humans have also been correlated with protection of subjects from viral infection^9–13^. It is worth noting that in a recent study with controlled human influenza virus challenge, when compared with HI antibodies, the levels of pre-existing NI antibodies correlated significantly better with respect to reduced disease severity score, decreased symptoms and a shortened duration of virus shedding^14^.

While the role of NA-induced immune response is unquestionable, the levels of NA-specific antibodies are only modest in people immunized with the standard influenza vaccine containing 15 μg of the hemagglutinin of each strain; yet, an eight-fold increase in the levels of NA-specific antibody was detected when four times concentrated standard vaccines were used, suggesting an increased amount of the NA antigen in vaccine can ensure a better immune response to NA^11^. These data reinforce the notion that the NA antigens in the influenza vaccines could be increased and better controlled so as to elicit a balanced immune response^15–17^. However, the neuraminidase in the current influenza vaccines is not quantified but only verified for the presence of enzymatic activity. Furthermore, the activity of neuraminidase as a potential potency marker is deemed unreliable since the enzymatic activity levels vary in the vaccine preparations or could be very low or even missing in some of them^17–19^. The variable level of enzymatic activity is likely due to the instability of the protein to the chemical treatments and storage procedures used in vaccine preparations^19–21^, making the determination of enzymatic activity unacceptable as a means for accurate quantification of NA in vaccines^22^.

A few alternative approaches have been explored to quantify NA in vaccines. Specifically, the use of strain specific antibodies in ELISA and immunochromatography has been reported^20,21^. However, these antibodies are strain specific and need to be made for the NA of each viral strain, which in practice would incur significant challenges and resources due to the rapid mutation rate of NA. Although physicochemical methods can quantify the HA proteins without the use of antibody-based reagents^23–32^ there has been no report on the quantitative detection of the NA proteins in trivalent vaccines with the exception of isotope dilution mass spectrometry (IDMS), which was used to analyse the NA activity in an H1N1 vaccine^33^. Clearly, lack of appropriate reagents or methods could have significantly impeded NA quantification^22^.

In a previous study, we developed a universal antibody against the most conserved epitope (222–230) of all influenza NA^34^. As the epitope targeted by the universal antibody is nearly 100% conserved in all NAs of both type A and type B virus, immunoassays based on this universal antibody can be used to quantify monovalent vaccine. Understandably, this NA universal antibody is not suitable for trivalent vaccines because the antibody would bind to all NA proteins in the vaccine preparations. To address the challenge in quantifying NA in trivalent vaccines, we set out to develop type/subtype-specific universal antibodies targeting the most conserved sequences in N1, N2 and NB, respectively. We report here that quantitative immunoassays based on these antibodies could be successfully used to analyze NA antigen contents in both monovalent and trivalent vaccines.

## Results

### Selection of peptides and generation of antibodies against N1, N2 and NB

Following our analysis of the public database, a set of 15 amino acid consensus sequences were identified for each subtype in a procedure used to maximize conservation and surface accessibility as described previously^35,36^. Sequence *QHPELTGLDCIRPCF (408–422)*, designated as CNA1, was selected for N1, *RTLLMNELGVPFHLG* (*159–173*), designated as CNA2, for N2, and *STFQKALLISPHRFG (90–104)*, designated as CNAB, for NB (N2 numbering). These identified peptides for N1, N2 or NB are remarkably conserved within their respective groups, i.e., CNA1 was found to be 94% conserved within N1 subtype, CNA2 97% for N2 subtype, and CNAB 99.79% for all type B neuraminidase. Importantly, these peptides share no similarity to other groups except their own.

The selected peptides were synthesized, modified with a 6-aminocaproic-cysteine linker and conjugated to a KLH carrier protein before used to immunize rabbits to produce mono-specific antibodies^35^.

### Preparation and Characterization of Antibodies

The mono-specific antibodies against CAN1, CAN2 and CANB were obtained after 4 rounds of immunization of rabbits. We first attempted to use an indirect ELISA by coating the plates with vaccine samples to determine Ab titers, yet no NA antibody was detected, which is likely due to that the amount of NA antigens in the vaccines being too low to be detected. To overcome this problem, GST fusion protein expressing the epitopes were constructed and used as the coating antigen in ELISA. The titer of all three antibodies against fusion proteins were above 1: 100,000 (Supplementary Fig. S1).

**Figure 1:**
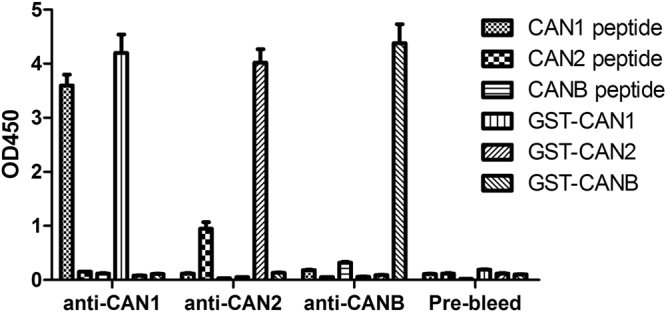
Binding of mono-specific antibodies to the peptides and fusion proteins. Peptides or GST-fusion proteins were coated onto 96-well plates, followed by incubation with the antibodies and subsequently detection with HRP-conjugated secondary antibodies.

We next determined the specificity of the monospecific antibodies. GST-fusion proteins or peptides were used as the coating antigen in an indirect ELISA. As shown in Fig. 1, CAN1, CAN2 and CAN/B monospecific antibodies bound strongly to their respective epitopes in the GST fusion proteins; the binding between the antibodies and peptide epitopes in GST-fusion protein is generally better than that between the antibodies and the unconjugated free peptides (Fig. 1). This observation is likely due to the unconjugated peptides having their epitopes masked upon coating to the plates.

### Binding of the antibody to the diverse strains/type of influenza viruses

**Figure 2:**
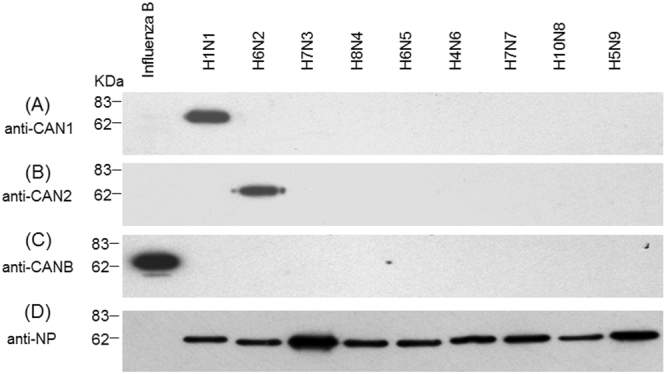
Binding of CAN1/2/B Antibodies to various type/subtypes NA proteins. Allantoic fluids containing viruses were fractionated in SDS-PAGE. Four gels were run and transferred to 4 membranes in parallel, followed by detection of the NA proteins using the CAN1 antibodies (panel A), CAN2 antibodies (panel B), CANB antibodies (panel C). Rabbit polyclonal anti-NP (nucleoprotein) proteins of influenza A viruses were used as control (panel D). Note: Anti-NP antibody against type A cannot bind to the NP of type B viruses. Original pictures see Supplementary Figure S3.

In order to determine whether the mono-specific antibodies exclusively bind to corresponding type/subtype of NA, WB assays were performed using all nine subtype of type NA and one type B NA. Allantoic fluids containing the viruses were used directly to determine the specificity of antibodies against the viral sequences. As shown in Fig. 2, all three antibodies can bind to their corresponding type/subtype of NA, with no significant cross-reactivity to any other type of NA or the allantoic fluid proteins.

**Figure 3:**
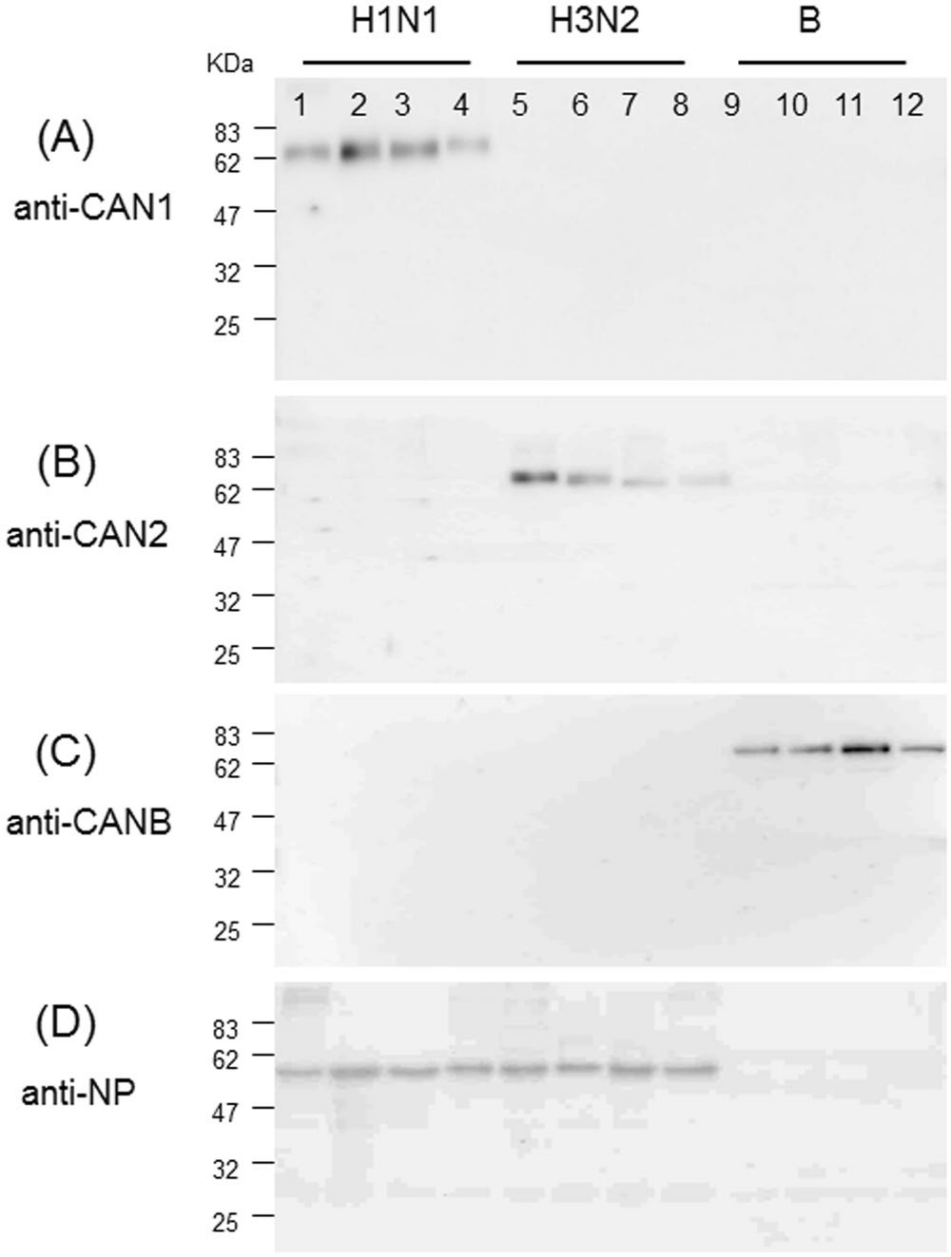
Binding of subtype-specific antibodies to their respective reference antigens derived from multiple viral strains. The reference antigens used are described in Table S1. Lane 1–4: 4 strains of H1N1; Lane 5–8: 4 strains of H3N2; Lane 9–12: 4 strains of type B vaccines. The samples were fractionated on SDS-PAGE (1 μg HA/lane). Four gels were run and transferred to 4 membranes in parallel, followed by detection of the NA proteins in W.B. using the CAN1 (panel A) and CAN2 antibodies (panel B) and CANB antibodies (panel C). Rabbit polyclonal anti-NP (nucleoprotein) proteins of influenza A viruses were used as control (panel D). Note: Anti-NP antibody against type A cannot bind to the NP of type B viruses.

We next analyzed binding of the antibodies to influenza reference antigens used for the potency determination of commercial influenza vaccines. In agreement with results of antibody binding to the viruses presented in Fig. 2, all three antibodies demonstrated type/subtype-specificity when four reference antigens were tested (Fig. 3). Taken together, these results indicate that these antibodies are highly specific for the viral NA sequences within their respective type/subtype.

### Antibody-based assay for NA quantification of products from eight manufacturers

Having succeeded in determining the type/subtype specificity of the mono-specific antibodies, we next investigated whether these antibodies could be used to quantitatively analyze the contents of NA in vaccine preparations. We employed a slot blot for NA quantification as this method has been shown to be simple assay capable of quantifying HA in a high throughput manner^37^.

We first analyzed the specificity of the NA subtype-specific antibody-based slot blot. To this end, vaccine reference antigens of H1N1 (A/California/7/2009), H3N2 (A/Hong Kong/4801/2014) and B (B/Brisbane/60/2008) were loaded on the membrane based on the HA concentration of 2 μg/ml. CAN1, CAN2 and CANB antibodies were added to react with the blotted proteins; we found that CAN1 could only bind to H1N1, CAN2 to H3N2 and CANB to type B vaccine, a result in agreement with that obtained by WB as described in Figs 2 and 3 (supplement information Fig. S2).

**Figure 4:**
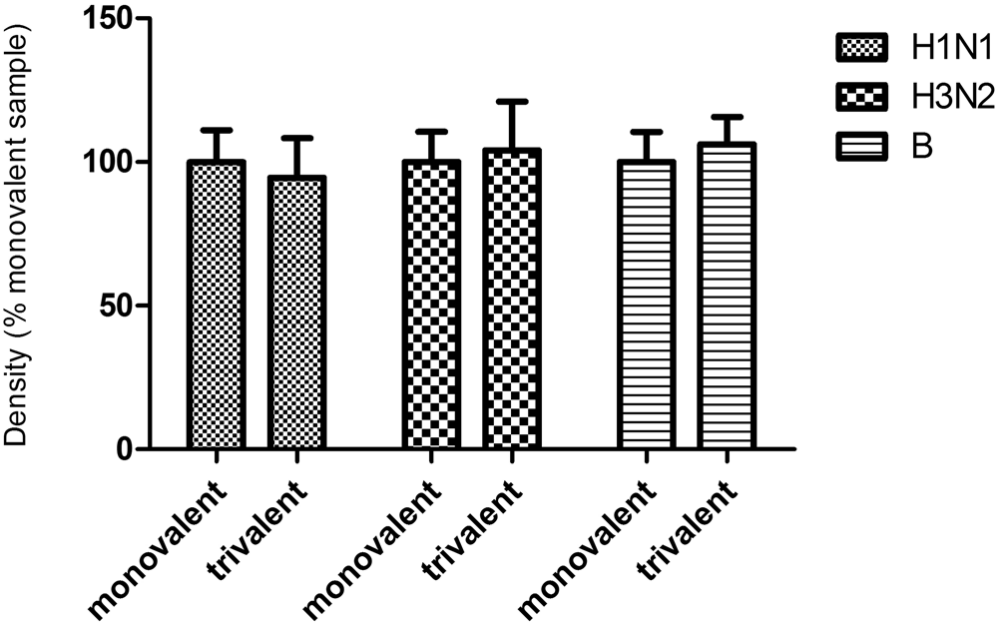
Specificity and interference of NA slot-blot. Samples were diluted to final concentration of HA 2 μg/ml (trivalent samples contain 2 μg/ml HA from each strain). Each sample was tested in six replicates. The NA contents of trivalent samples were normalized and reported as percentage of monovalent samples.

Because current vaccines are mainly trivalent formulations, we next determined potential interference in these final products. Monovalent and trivalent preparations (2 μg/ml HA from each strain) were compared in the slot blot. As shown in Fig. 4, the NA antigens in monovalent and trivalent detected with their respective NA antibodies were comparable, indicating that presence of three different NA antigens did not interfere with the ability of antibodies in binding to their respective NA.

**Figure 5:**
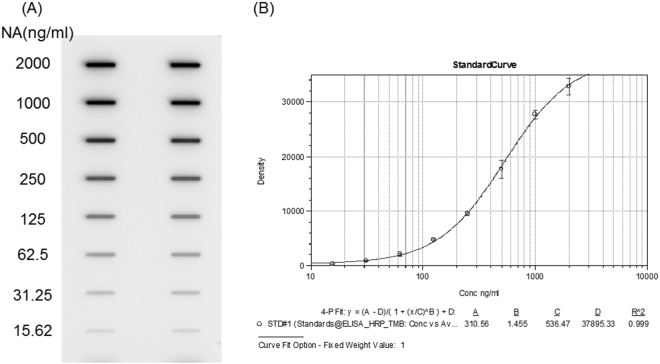
Development of standard curve for NA quantification using slot blot. Panel A: quantitative detection of NA using CAN1 antibodies against N1: the N1 antigens at various concentrations were loaded onto NC membrane. The membrane was then incubated with the CAN1 antibodies, followed by detection with anti-rabbit IgG peroxidase conjugate. Similar results were obtained with CAN2 and CANB antibody binding to their corresponding NA antigens (not shown in the figure). Panel B: standard curve for the quantification of N1: the currently accepted 4-parameter logistic (4-PL) model was employed for the calibration curve fitting in the immunoassays. Original pictures see Supplementary Figure S4.

We next developed the standard curve using the reference antigens for quantitative analyses of vaccine samples. As presented in Fig. 5A, the concentration of the NA antigen references from H1N1 was proportional to the signal intensity developed in slot blot, with curve fitting very well with the 4-PL model (R^2^ = 0.99) as shown in Fig. 5B. Therefore, the standard curve was used for subsequent analyses of vaccine samples.

To assess the reproducibility, three lots vaccine preparations were analyzed in three separate experiments and results for the measured potencies were compared. As presented in Table 1, little variation was observed between runs, as demonstrated by the low standard deviation values and the coefficient of variance values between 2.4% and 14.3%.

**Table 1:** Reproducibility of the slot blot assay.

	Samples	Average of three runs (ng/ml ± SD)	CoV(%)	95%CI
H1N1	batch1	5,325 ± 527	9.9%	4,016–6,634
	batch2	5,148 ± 237	4.6%	4,559–5,737
	batch3	3,335 ± 477	14.3%	2,150–4,520
H3N2	batch1	1,942 ± 56	2.9%	1,803–2,081
	batch2	1,320 ± 137	10.4%	980–1,660
	batch3	929 ± 22	2.4%	874–984
B	batch1	164 ± 10	6.1%	139–189
	batch2	183 ± 8	4.4%	163–203
	batch3	281 ± 13	4.6%	249–313

Note: The result of each batch was obtained from three runs of test.

Having developed the standard curve for N1, N2 and NB quantification, we next analyzed NA antigens in eight commercial influenza vaccines for the 2016–2017 flu season. We found that the NA levels vary dramatically among N1, N2 and NB, with N1 being the highest and NB the lowest no matter which manufacturers’ products were analyzed (Table 2). Although the NA levels could be relatively close in several lots of products from a given manufacturer, the amount of NA varies drastically between the eight different manufacturers. For example, NA amounts of H1N1 in manufacturer A were consistently higher than other seven producers’ products whereas manufactures E, F and H have lower NA amounts, with F and H generally being the lowest. This is true regardless N1, N2 or NB was compared. Interestingly, we were able to obtain six vaccine lots from manufacturer A for NA quantification; we found NA contents, irrespective of the vaccine strains (H1N1, H3N2 and Type B), were fairly consistent and generally higher than the vaccines from other manufacturers, particularly for N1 of H1N1 which is more than 5 μg/ml or roughly one-fifth of the HA amounts. This ratio of 1/5 between NA and HA approaches the expected ratio of 1:4 in some native viral strains^38^. As only two or three lots were analyzed from other producers, it is hard for us to draw any conclusion even though it appears that the variations in NA levels were less pronounced than that between the manufacturers. However, compared to producers A or D, F and H have nearly 10–20 times less NA in their products or NA/HA ratio was only 1/100–2/100, far below the theoretical ratio of NA/HA (25/100) in an influenza virus^38^. Taken together, these data indicate that manufacturing process could dramatically affect the levels of NA in the current influenza vaccines.

**Table 2:** NA contents in 2016~2017 seasonal influenza vaccines.

Manufactures	Lots	Average ng NA/ml ± SD		
		H1N1	H3N2	B
A	A1	5,703 ± 735	1,147 ± 23	210 ± 8
	A2	6,080 ± 710	1,197 ± 235	452 ± 37
	A3	5,284 ± 1,038	1,458 ± 113	188 ± 24
	A4	5,664 ± 983	1,647 ± 79	183 ± 4
	A5	5,361 ± 824	1,409 ± 116	215 ± 7
	A6	5,142 ± 1,074	1,403 ± 47	172 ± 7
B	B1	1,447 ± 88	774 ± 39	<32
	B2	1,673 ± 49	428 ± 44	<32
	B3	1,975 ± 242	819 ± 63	<32
C	C1	2,628 ± 5	1,797 ± 128	<32
	C2	2,147 ± 353	1,350 ± 179	<32
	C3	2,137 ± 460	1,316 ± 361	<32
D	D1	4,654 ± 519	1,375 ± 156	320 ± 20
	D2	4,441 ± 213	1,323 ± 35	414 ± 86
	D3	3,848 ± 248	1,262 ± 118	347 ± 34
E	E1	1,307 ± 307	690 ± 39	93 ± 16
	E2	1,151 ± 131	600 ± 28	100 ± 27
	E3	1,201 ± 247	704 ± 24	100 ± 23
F	F1	603 ± 76	<32	<32
	F2	401 ± 35	<32	244 ± 51
G	G1	3,797 ± 1,002	1,036 ± 33	265 ± 4
	G2	3,261 ± 740	747 ± 17	215 ± 7
H	H1	145 ± 18	<32	<32
	H2	243 ± 144	<32	<32
	H3	200 ± 82	<32	<32

Note: the HA antigen content of 3 subtype/type are within 30~36 μg/ml; each sample was tested three times.

## Discussion

HA and NA are two major surface proteins on influenza virus particles, both of which are well known to elicit a protective immune response. However, only HA is quantitatively determined and serve as the potency marker in the current inactivated influenza vaccines, whereas NA is not quantified but only verified for the presence of neuraminidase activity in vaccine bulks. The measurement NA activity is unreliable for quantitative determination of NA as its activity could be dramatically affected in the manufacturing process such as chemical treatment, e.g. formalin inactivation and ether treatment for virus splitting. Clearly, a simple method capable of quantifying the NA proteins would be of great use for vaccine quality control.

In our previous report, two highly conserved regions in virtually all influenza viruses NA were identified through extensive bioinformatics analyses, one of which was found to be a nearly 100% conserved among all type A and B influenza strains. Universal antibodies against this epitope could bind all nine subtypes of NA in type A as well as all NA in type B^34^. Although the universal anti-NA antibodies could be used to quantify monovalent vaccines or used for in-process control, they cannot be used to determine NA antigens in the current trivalent or quadrivalent vaccines. Thereby, it would be essential to develop an immunoassay capable of quantitatively differentiating the NA type and subtypes in the current seasonal vaccines.

Several lines of evidences prompted us to develop this NA quantification assay. First, like HA, NA is also known to be evolving in an unpredictable fashion, making it necessary to develop antibodies which require no need for annual updating. Second, the antibodies must be able to distinguish NAs in the trivalent vaccines. Third, the NA should be determined by a simple assay in a high throughput manner. Fourth, there has been no report on the analyses of NA variations in trivalent vaccines from various manufacturers.

To achieve these goals, we first conducted comprehensive bioinformatics analyses of all NA strains in the public database. Interestingly, all consensus sequences within NA subtypes found by Shannon entropy analysis are located in the head region of NA, which is formed by six identical antiparallel β-sheets (motifs) organized in the form of a propeller-like structure. Although the conserved sequences of both N1 and N2 subtype were successfully identified on motif 1, 2 and 6, respectively, only those sequences from motif 6 of N1 and sequences from motif 1 of N2 were found to be dissimilar with other subtypes, an observation further verified by multiple alignment and Shannon entropy analysis. Thus, the epitope with the greatest differential abundance (i.e. conserved in each of N1, N2 and NB, but absent in other NA subtypes) was finally selected. Specifically, amino acid 408–422 was chosen for N1, amino acid 159–173 for N2 and amino acid 90–104 for NB. Indeed, mono-specific antibodies targeting these peptides were confirmed in subsequent experiments to bind to their corresponding NA type/subtype only and have no cross reactivity to other subtype NA or allantoic proteins.

The potential of the NA antibody-based assay for NA quantification was explored using eight manufacturers’ products. While the variation of NA in different virus strains is not surprising, drastic difference of NA in products from eight manufacturers is noteworthy as they use the same seed strains provided by the WHO for vaccine production. Some products consistently have NA/HA ratio close to that in a native virus (1:4)^38^, whereas others contain 10–20 fold less of NA in the final products. It remains unclear as to how the NA proteins were lost during the manufacturing process, given each producer may have different manufacturing process. Hopefully, with simple detection method like this, it could be easier to conduct in-process control. Nevertheless, these findings reveal that manufacturing process can significantly influence NA amounts, and it might be worth considering incorporation of this type of assay for better quality control. Unlike the HA antibodies which need to be annually updated, the NA antibodies reported here may be of practical value for vaccine developers, industry and regulatory agencies.

Finally, it is worthy to mention that although there are mounting evidences supporting the role of NA in inducing protective immune responses, HA remains to be the critical antigens. Indeed, recombinant HA vaccine products have been licensed for human vaccination^39^. While the amount of HA per dose in the recombinant vaccine (45 µg) is three time more than that in the egg-based inactivated vaccine (15 µg), the difference in the HA amounts between rHA and current inactivated vaccine remains to be fully understood in terms of induction of immune responses. Moreover, other forms of vaccines are also being explored including virus-like particle-based vaccines; the VLPs under development are reported to vary in the compositions of the viral proteins^40,41^. Future work should be conducted to determine whether incorporation of NA in these new generations of products may improve the immunogenicity of these products. The availability of tools for NA quantification could facilitate the development of newer generations of vaccines. Interested investigators may contact the authors to request the antibody reagents for their research work.

## Materials and Methods

### Viruses and vaccines

Table S1 lists the viruses, human vaccines and vaccine reference antigens used in the characterization of the antibodies. All the virus strains used in this study were the same as reported previously^34^. The viruses were propagated in embryonated chicken eggs. The flu vaccine reference antigens were kindly provided by the National Institute for Biological Standards and Control (NIBSC), Potters Barr, UK and Therapeutic Goods Administration (TGA), Symonston ACT 2609, Australia.

The seasonal influenza vaccines used in this study are derived from 3 virus strains recommended by WHO for the 2016–2017 Northern Hemisphere, i.e., A/California/7/2009(H1N1), A/Hong Kong/4801/2014(H3N2) and B/Brisbane/60/2008 (B).

### Identify the conserved but unique epitopes in NA type/subtype

To identify universally conserved regions specific to the N1, N2 or NB, a bioinformatics approach was employed as previously described^35^. In brief, all NA sequences from NCBI influenza virus resource (http://www.ncbi.nih.gov/ genomes/FLU/FLU.htm) were downloaded separately for each NA subtype and NB, with redundant or identical sequences deleted. A multiple alignment was then performed for each subtype followed by extraction of the consensus sequences from full-gene genome, in which the conservation index were sorted by the degree of variation and determined by calculating the Shannon entropy for each position of amino acid^35^. Geneious 7.0.6 software was then used to confirm the bioinformatics findings and ensure the uniqueness in one subtype and universality of the selected peptide within its own subtype^36^.

### Preparation of peptides and their conjugates for immunization

The current trivalent inactivated influenza vaccines are derived from one strain of A/H1N1, A/H3N2 and B type of influenza virus, respectively. Based on bioinformatics analyses, one peptide was chosen for each of the three groups N1, N2 and NB (more details in Results). These peptides were synthesized, modified with a 6-aminocaproic-cysteine linker and conjugated to a KLH carrier protein; the peptide-conjugates were then used to immunize rabbits for the preparation of type/subtype-specific universal antibodies as previously described^35^.

### Expression of fusion proteins containing epitopes

The GST fusion proteins containing each of CAN1, CAN2 and CANB epitopes were prepared for characterization of the antibodies. In short, forward and reverse oligonucleotides (Table S2) were synthesized according to the sequence of the peptide. After annealing, the double stranded polynucleotides were inserted into the pET19b-GST vector using the BamH1/Xho1 restriction sites. The pET19b-GST-CAN1/2/B recombinant plasmids were transformed into competent Escherichia coli BL21 (DE3) to express proteins. The bacterially expressed proteins were purified by His Gel (GE Healthcare, Uppsala, Sweden) according to the manufacturer’s instructions.

### ELISA

96-well plates (Costar, Kennebunk, ME) were coated at 4 °C overnight with 100 μl/well of CAN1, CAN2 or CANB peptides or their respective fusion proteins at 1 μg/ml in 0.01 M bicarbonate buffer (pH 9.6). The plates were washed 5 times by PBS-T, followed by blocking with PBS containing 5% skim milk for 1 h at room temperature. The type/subtype specific universal antibodies was then added, followed by incubation for 1 h at room temperature before HRP conjugated goat anti rabbit IgG antibody was added. After incubation for 1 h at room temperature, the plates were washed 5 times before Tetramethylbenzidine (TMB) substrate was added for colorimetric development. Optical density at 450 nm was measured by a Multiskan Ascent microplate reader (Thermo Scientific, Shanghai, China). Unless specified, pre-immunization serum was used as negative control.

### Western blot

NA antigens from all nine subtypes of type A influenza virus and Type B influenza virus were used to determine the type/subtype specificity of the NA antibodies by Western blot (WB). In brief, all allantoic fluids directly from eggs inoculated with influenza viruses were fractionated on sodium dodecyl sulfate (SDS)-12% polyacrylamide gels (PAGE), followed by transferring the samples to PVDF membrane (Millipore, Beijing, China). The membrane was then blocked with 5% skim milk in PBS at 37 °C for 1 h, followed by incubation for 1 h at 37 °C with NA-antisera as described above. Peroxidase-conjugated goat anti-rabbit immunoglobulin(IgG) (Sigma, St. Louis, MO) was added for an additional incubation of 1 h at room temperature, followed by chemiluminescent detection using ECL prime (GE health, Buckinghamshire, UK).

### NA antigen reference preparation

The NA reference materials were prepared by SDS-PAGE as reported previously^42^. In brief, a monovalent bulk material was treated with glycosidase PNGase F (New England Biolabs, Ipswich, MA) at 37 °C for 16 h in the buffer provided by the manufacturer in the presence of 1% NP 40; the samples were then subjected to SDS-PAGE separation. The position of NA band in SDS-PAGE was confirmed by mass spectrometry. Protein quantification were conducted by densitometric analyses of the protein bands using a high resolution scanner and Launch Doc-ITLS software (for UVP Bio Doc-It Imaging System, Upland. CA, USA). The amounts of NA were determined based on the ratio of NA protein to the total proteins^42^.

### Slot blot

Slot blot was conducted as described previously^36^. Briefly, the vaccines and the NA references were treated with 4 M urea (final concentration) to dissolve the aggregates which could form during the storage. The NA reference antigens in serial dilutions ranging from 15 to 2000 ng/ml were used to make a standard curve. Vaccine samples were tested in duplicate. After the reference materials and samples were loaded on membrane, type/subtype-specific antibodies were used as primary Ab incubation, followed by a 45 min incubation at room temperature with a 1/15,000 dilution of the ECL HRP-conjugated sheep anti-rabbit IgG in5% milk/TBS-Tween 0.1% buffer. After the addition of the SuperSignal West Dura Extended Duration substrate for 5 min, the blots were exposed for 10 to 60 s in a Bio-Rad ChemiDocTMMPImaging System. The ImageLab 4.0 software was used to measure the density of the bands.

### Data analysis

Unless specified, results are presented as mean ± SD with *n* = 3 and the currently accepted 4-parameter logistic (4-PL) model was employed for the calibration curve fitting in the immunoassays by SoftMax Pro v5 (Molecular Device, Sunnyvale, CA).

### Data availability statement

Any materials, data and associated protocols in this publication would be promptly available to readers without undue qualifications in material transfer agreements. The antibody reagents reported in this paper will be available to scientific community for research use only.

## Electronic supplementary material

Supplementary information
