# Supplementary material for: Universal type/subtype-specific antibodies for quantitative analyses of neuraminidase in trivalent influenza vaccines
Source: Sci Rep. 2018 Jan 18;8:1067. doi: 10.1038/s41598-017-18663-6 (PMC5773574; doi:10.1038/s41598-017-18663-6)
Supplement: Supplementary file 1 — Supplementary information [file 41598_2017_18663_MOESM1_ESM.pdf]

Universal type/subtype-specific antibodies for quantitative analyses of neuraminidase in trivalent influenza vaccines

Kangwei Xu[1,2#], Changgui Li[2#],Caroline Gravel[3], Zheng Jiang[2], Bozena Jaentschke[3], Gary Van Domselaar[4]*, Xuguang Li[3,5]*, Junzhi Wang[1,2]*

1. The State Key Laboratory of Cancer Biology, Department of Biochemistry and Molecular Biology, The Fourth Military Medical University, Xi'an, Shaanxi 710032, P.R. China
2. National Institutes for Food and Drug Control and WHO Collaborating Center for Standardization and Evaluation of Biologicals, Beijing, 102619. P.R. China
3. Center for Biologics Evaluation of Biologicals, Ottawa, Biologics and Genetic Therapies Directorate, Health Canada and WHO Collaborating Center for Standardization and Evaluation, Ottawa, ON K1A 0K2, Canada
4. National Microbiology Laboratory, Public Health Agency of Canada, Winnipeg, MB R3E 3R2, Canada
5. Department of Biochemistry, Microbiology and Immunology, Faculty of Medicine, University of Ottawa, Ottawa, ON, Canada

Running title:

Subtype-specific universal antibodies against neuraminidase

# These authors contributed equally to the work.

*Corresponding Authors: GVD (gary.vandomselaar@phac-aspc.gc.ca), XL

(sean.li@hc-sc.gc.ca), JW (wangjz@nifdc.org.cn)

Supplementary Figure S1: ELISA titer of anti-serum against fusion proteins. The CAN1, CAN2 and CANB peptides conjugated to KLH through a Acp-KKC linker were injected into rabbits subcutaneously and boosted every two week. Indirect ELISA with fusion proteins as coating antigen were used to measure the antibody titer of corresponding anti-serum. Note: n = 3, with error bars indicating SD.

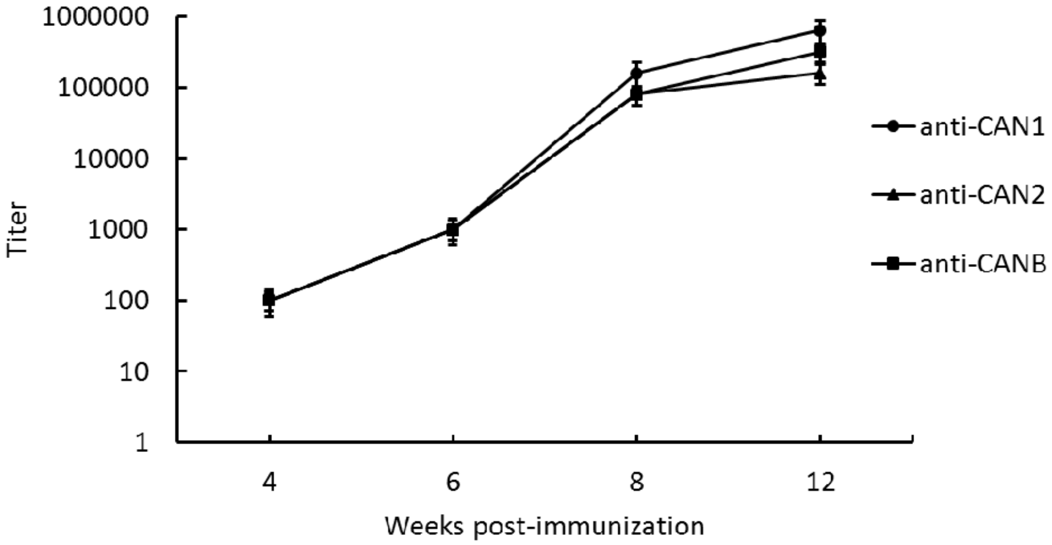

Supplementary Figure S2: Specificity of subtype-specific NA antibodies in slot-blot. Vaccine reference antigens of H1N1 (A/California/7/2009), H3N2 (A/Hong Kong/4801/2014) and B (B/Brisbane/60 /2008) were diluted to final concentration of HA 2µg/ml, loaded onto NC membrane. The membrane was then incubated with the antibodies respectively, followed by detection with anti-rabbit IgG peroxidase conjugate.

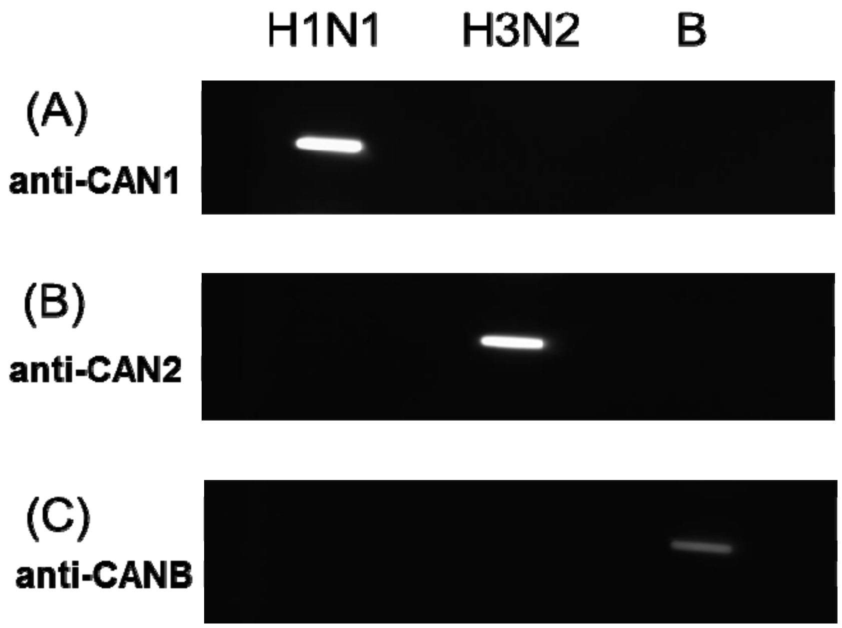

Supplementary Figure S3: Original Blot Pictures of Figure 2

For Figure 2A

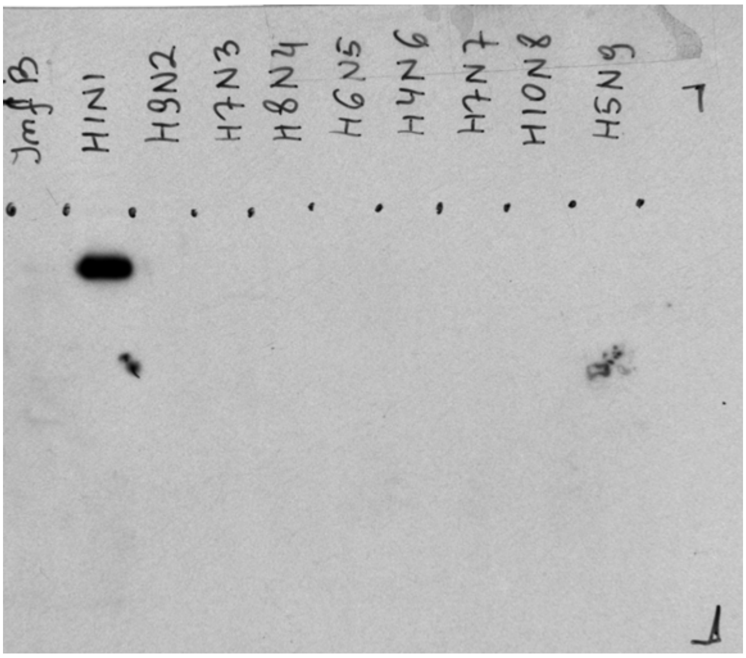

For Figure 2B

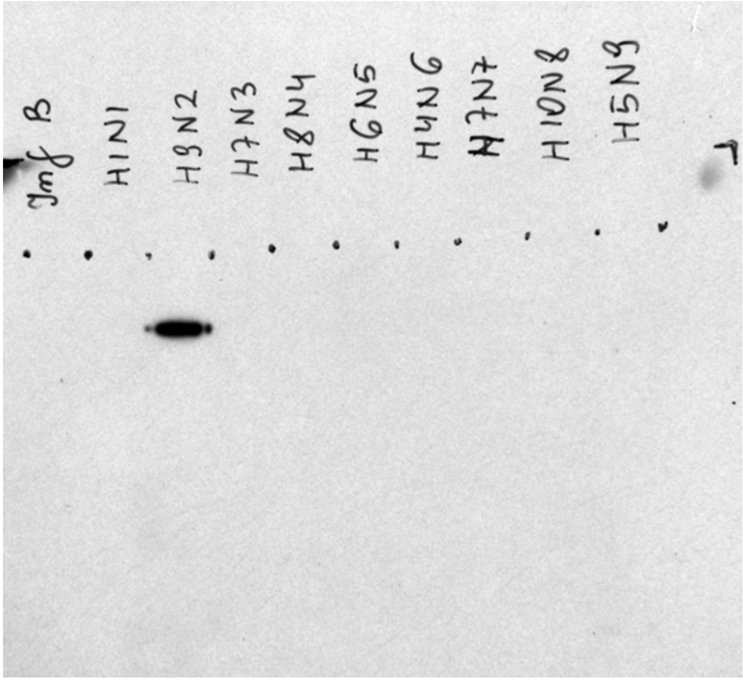

For Figure 2C

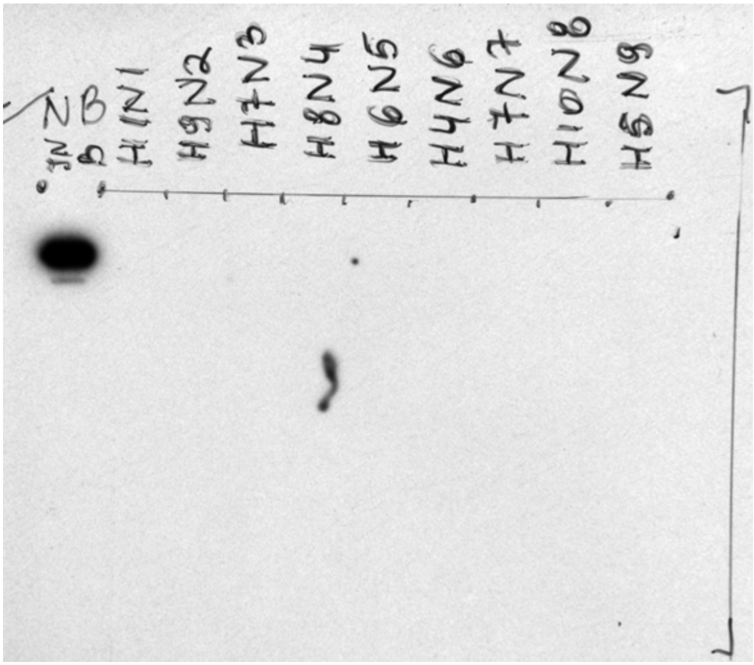

For Figure 2D

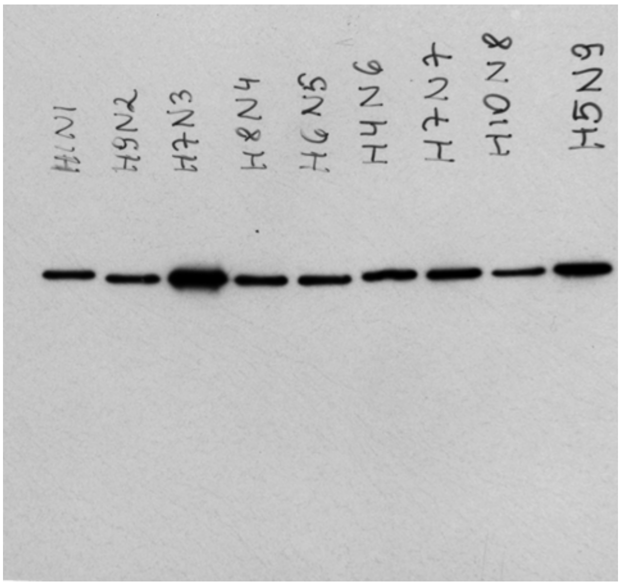

Supplementary Figure S4: Original Blot Picture of Fig.5A

In this experiment, the NA standards from 15.62 to 2000ng/ml and vaccine samples were run together. The cropped NA standards were used for Fig.5A.

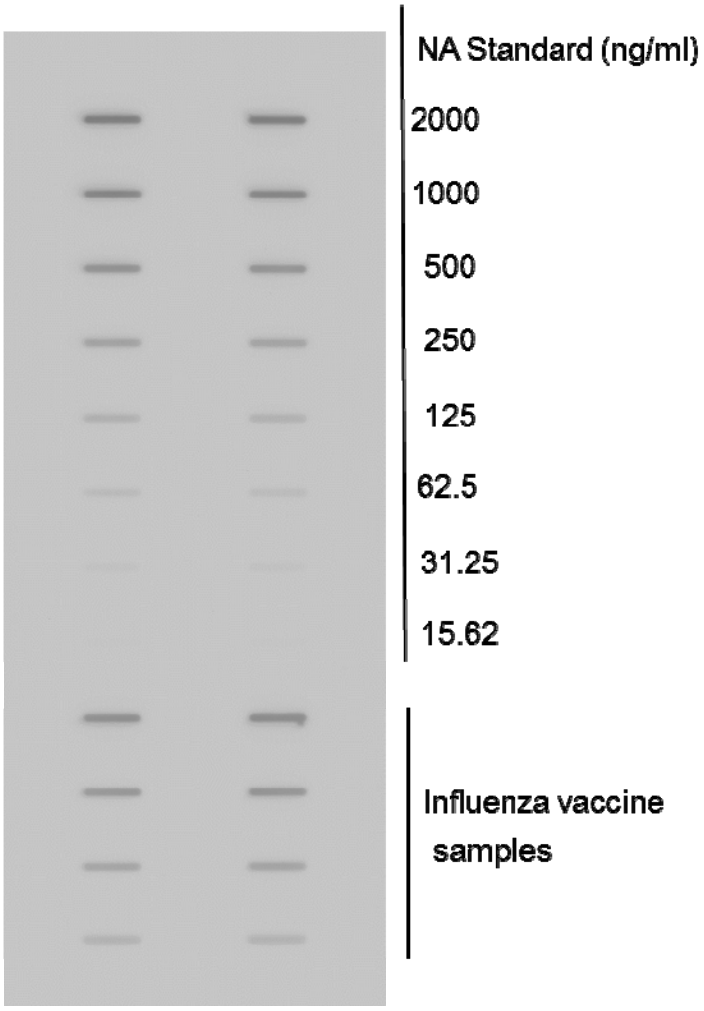

Supplementary Table S1: Influenza virus and vaccine HA reference antigen used in the studies

Influenza virus	
Subtype	Strains
H1N1	A/duck/Shantou/1734/2003
H9N2	A/Turkey/Mass/3740/65
H7N3	A/Turkey/Oregon/71
H8N4	A/turkey/Ontario/6118/1968
H6N5	A/Shearwater/Australia/72
H4N6	A/DK/Czechoslovakia/56
H7N7	A/EQ/Prague/1/56
H10N8	A/Quail/Italy/1117/65
H5N9	A/turkey/Wisconsin/68
B	B/Singapore/222/97

Influenza vaccine HA referance antigen			
Subtype	Strains	Source	Code
H1N1	A/New Caledonia/20/99	NIBSC	03/258
H1N1	A/Brisbane/59/2007	NIBSC	08/100
H1N1	A/New Caledonia/20/99	NIBSC	04/256
H1N1	A/California/7/2009	NIBSC	09/146
H3N2	A/WYOMING/03/03	NIBSC	03/220
H3N2	A/NEW YORK/55/2004	NIBSC	04/264
H3N2	A/Brisbane/10/2007	NIBSC	08/124
H3N2	A./Victoria/210/09	NIBSC	10/102
B	B/Jiangsu/10/2003	NIBSC	04/202
B	B/Brisbane/3/2007	TGA	2007/80B
B	B/Florida/4/2006	NIBSC	07/262
B	B/Brisbane/60/2008	TGA	2009/82B

Supplementary Table S2: Oligonucleotides used for fusion proteins express

Name	Peptide sequence N→C	Oligonucleotides sequence 5'→3'
CAN1	QHPELTGLDCIRPCF	GATCCCAGCATCCGGAACTGACCGGCCTGG ATTGCATTCGCCCGTGCTTTTAAC(sense)
		TCGAGTTAAAAGCACGGGCGAATGCAATCCA GGCCGGTCAGTTCCGGATGCTGG(antisense)
CAN2	RTLLMNELGVPFHLG	GATCCCGCACCCTGCTGATGAACGAACTGGG CGTGCCGTTTCATCTGGGCTAAC(sense)
		TCGAGTTAGCCCAGATGAAACGGCACGCCCA GTTCGTTCATCAGCAGGGTGCGG(antisense)
CANB	STFQKALLISPHRFG	GATCCTCTACCTTTCAGAAAGCGCTGCTGATTT CTCCGCATCGCTTTGGCTAAC(sense)
		TCGAGTTAGCCAAAGCGATGCGGAGAAATCAG CAGCGCTTTCTGAAAGGTAGAG(antisense)
